# Transforming Digital Phenotyping Raw Data Into Actionable Biomarkers, Quality Metrics, and Data Visualizations Using Cortex Software Package: Tutorial

**DOI:** 10.2196/58502

**Published:** 2024-08-23

**Authors:** James Burns, Kelly Chen, Matthew Flathers, Danielle Currey, Natalia Macrynikola, Aditya Vaidyam, Carsten Langholm, Ian Barnett, Andrew (Jin Soo) Byun, Erlend Lane, John Torous

**Affiliations:** 1 Division of Digital Psychiatry Beth Israel Deaconess Medical Center Harvard Medical School Boston, MA United States; 2 Case Western Reserve University School of Medicine, Cleveland, OH United States; 3 Carle Illinois College of Medicine Urbana, IL United States; 4 Department of Biostatistics Epidemiology and Informatics Perelman School of Medicine at the University of Pennsylvania Philadephia, PA United States

**Keywords:** digital phenotyping, mental health, data visualization, data analysis, smartphones, smartphone, Cortex, open-source, data processing, mindLAMP, app, apps, data set, clinical, real world, methodology, mobile phone

## Abstract

As digital phenotyping, the capture of active and passive data from consumer devices such as smartphones, becomes more common, the need to properly process the data and derive replicable features from it has become paramount. Cortex is an open-source data processing pipeline for digital phenotyping data, optimized for use with the mindLAMP apps, which is used by nearly 100 research teams across the world. Cortex is designed to help teams (1) assess digital phenotyping data quality in real time, (2) derive replicable clinical features from the data, and (3) enable easy-to-share data visualizations. Cortex offers many options to work with digital phenotyping data, although some common approaches are likely of value to all teams using it. This paper highlights the reasoning, code, and example steps necessary to fully work with digital phenotyping data in a streamlined manner. Covering how to work with the data, assess its quality, derive features, and visualize findings, this paper is designed to offer the reader the knowledge and skills to apply toward analyzing any digital phenotyping data set. More specifically, the paper will teach the reader the ins and outs of the Cortex Python package. This includes background information on its interaction with the mindLAMP platform, some basic commands to learn what data can be pulled and how, and more advanced use of the package mixed with basic Python with the goal of creating a correlation matrix. After the tutorial, different use cases of Cortex are discussed, along with limitations. Toward highlighting clinical applications, this paper also provides 3 easy ways to implement examples of Cortex use in real-world settings. By understanding how to work with digital phenotyping data and providing ready-to-deploy code with Cortex, the paper aims to show how the new field of digital phenotyping can be both accessible to all and rigorous in methodology.

## Introduction

The popularity of smartphone-based digital phenotyping for advancing health research has resulted in a plethora of platforms and tools. Many have lauded the potential to capture real-time behavioral data from patients’ smartphones in a scalable manner [1,2]. However, recent reviews and commentaries consistently highlight the need for improved methodological rigor, more return of results to participants, and better data sharing [3-5]. Solving these challenges is multifactorial but can be framed around issues of (1) data collection, (2) data processing, and (3) return of results.

The evolving landscape of consumer technology, and thus digital phenotyping apps, presents numerous challenges to their scientific study. These apps require constant efforts to ensure they remain secure and able to collect the data they claim to capture. The result of this dynamic environment is that many digital phenotyping apps are no longer supported, and many past apps are already abandoned. Keeping pace with new updates from Apple and Google, security vulnerabilities or patches, user interface or experience improvements, and new sensors accessible from the phone requires constant effort and financial resources. Furthermore, the shifting tools and libraries used in this research has also created challenges for transparent data processing and return of results. Few digital phenotyping studies have ever been replicated because differences in feature processing procedures make the resulting outputs from these studies incomparable. Of note, in the rare cases in which replication has been attempted, the results have been negative [6,7].

Beyond supporting replicable science, better data collection and data processing can enable better data visualization, better sharing of results with participants, and meaningful use outside of research. A 2022 paper proposed the return of results as a necessary aspect of ethical digital psychiatry research [8], and a 2023 ethical framework was proposed that specially identified the need to return results in digital phenotyping in psychiatry [9]. However, neither proposal identified how this could be accomplished or provided resources to ensure data sharing is possible. The need for data visualizations extends beyond just research. A December 2023 systematic review and meta-analysis on the utility of mental health apps to augment care for psychiatric illnesses found that, while nearly two-thirds of those apps reviewed captured digital phenotyping–related data, less than half of these apps were even able to share data with clinicians [10]. A 2022 review on data visualization in mental health highlighted the numerous benefits of return of results for patients including enabling proactive self-management, more effective communication with clinicians, and better engagement with remote tools like apps [11].

**Figure 1:**
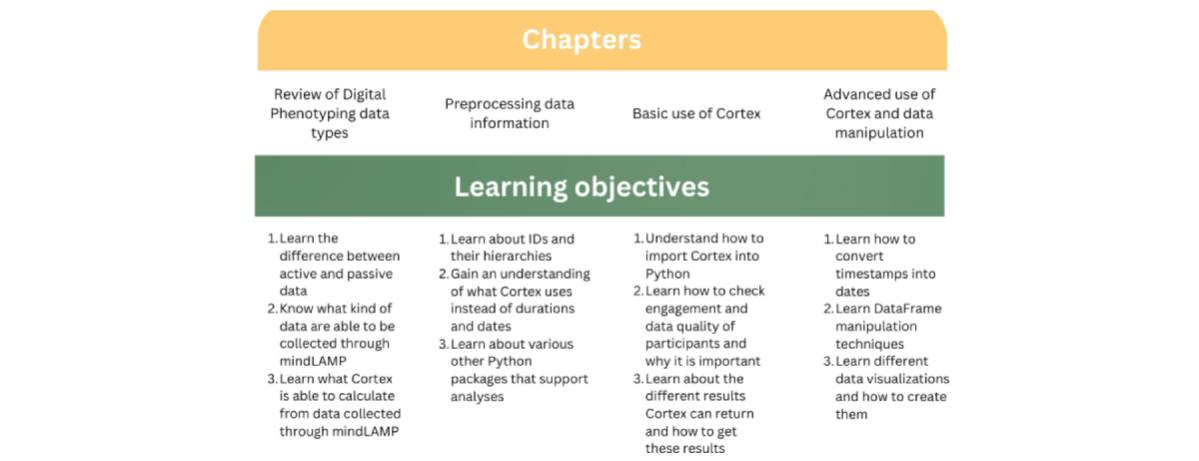
Paper schematic.

Toward supporting more transparent and ethical research, the need for better data collection, data processing, and return of results is clear. Data processing pipelines offer a productive focus, as they can support data from multiple apps and transform that data to support myriad visualizations. There are several pipelines specific to smartphone digital phenotyping data including Rapids [12], Forest [13], and Cortex [14]. While each of the platforms offers value, they also feature differences, especially around natively supporting data visualizations. Toward the goal of expanding the utility of digital phenotyping methods and enabling the return of results, we review the Cortex data processing pipeline and provide a tutorial to encourage more replicable, ethical, and clinically relevant research. Toward building the use cases of data visualization, this paper (1) reviews digital phenotyping data types, (2) explores preprocessing steps, (3) discusses the basic use of Cortex for data quality assessments, (4) presents advanced Cortex features, and (5) provides several real-world examples of using all the steps together. Figure 1 below provides a visual overview of the paper and details of the topics covered.

The goal of this paper is to facilitate an understanding of the steps, assumptions, and limitations around processing digital phenotyping data and transforming it into data visualizations. The steps outlined here correspond to interactive examples, and we highlight in each step the ability to customize, expand, or alter the procedures to ensure the process can serve a range of outcomes, from simple reporting of survey scores to more complex time series results. To facilitate these examples, we draw from the open-source mindLAMP application, which is used by nearly 100 research teams across the world [15-18].

### Review of Digital Phenotyping Data Types

Different types of digital phenotyping data require different approaches toward labeling, storing, and processing. In digital phenotyping and with mindLAMP, data are classified as either active or passive. Active data include surveys and cognitive assessments. These data streams are customizable by the researchers or clinician and require the patient or user to actively engage (ie, take a survey) for data to be collected. mindLAMP can be deployed solely as a survey tool, making it possible to use the platform for ecological momentary assessment research alone. All survey and cognitive data are stored within a secure server with no personal identifiable information; only the participant’s user ID is attached for identification [19].

By contrast, passive data are collected using the phone’s native sensors enabled by the participant and also specified by the researcher. These sensors commonly include an accelerometer and a GPS, both of which are assigned to collect at a frequency of up to 5 and 1 Hz, respectively. The passive data collected through sensors can be customized by a study to use as few or as many sensors as researchers desire, with options ranging from screen time to Bluetooth connections. It is also important to note that these sensor outputs are also referred to as raw data. They are what Cortex uses to calculate more actionable metrics. Table 1 offers a more comprehensive list of options at the time of this writing. Table 1 lists all the sensors LAMP currently has the ability to collect and their name within mindLAMP. Sampling type represents how much data are collected within a certain time frame. If the sampling type is continuous, all data are collected the moment the sensor is activated. If the sampling type is discrete, the data are recorded every *n* seconds, in which *n* is defined by a frequency parameter. If the sampling type is interval, which only relates to step count, the data describe how many steps were taken within *n* minutes, which is described by a frequency parameter.

Unlike active data, passive data are captured at a high volume and velocity that are not conducive to most ecological momentary assessment data analysis methods. It must be processed into actionable metrics through a pipeline such as Cortex. For example, Cortex can process thousands of coordinates from the GPS phone sensor and transform them into a metric that reflects how much time a participant spent at home, appropriately named “hometime.” Table 2 offers a more comprehensive list of calculated data features offered by Cortex at the time of this writing. Table 2 lists all the secondary features currently offered by Cortex within the “Secondary feature name” column. It also lists the reference citations that feature links to the documentations, which offer a description and an example (per link) of how to pull values using cortex.secondary.feature_name.feature name(). More information regarding the command can be found in the *Passive Data Processing* section. The final column, “Requires additional parameters for cortex?” has a Boolean (True or False) outcome that represents if Cortex needs any additional parameters beyond start, end, and resolution to run either cortex.secondary.feature_name.feature_name() or cortex.run().

**Table 1:** Sensor types.

Name	SensorSpec	Sampling type	Requires watch or other devices
Analytics	lamp.analytics	Continuous	No
Location	lamp.gps	Discrete	No
Accelerometer	lamp.accelerometer	Discrete	No
Device Motion	lamp.device_motion	Discrete	No
Screen	lamp.device_state	Continuous	No
Pedometer	lamp.steps	Interval	No
Bluetooth & WiFi	lamp.nearby_device	Discrete	No
Calls & Texts	lamp.telephony	Continuous	No
Sleep	lamp.sleep	Continuous	Yes
Workouts	lamp.segment	Continuous	Yes
Activity Recognition	lamp.activity_recognition	Continuous	Yes
Nutrition	lamp.nutrition	Continuous	Yes
Blood Glucose	lamp.blood_glucose	Continuous	Yes
Oxygen Saturation	lamp.oxygen_saturation	Continuous	Yes
Body Temperature	lamp.body_temperature	Continuous	Yes
Blood Pressure	lamp.blood_pressure	Continuous	Yes
Heart Rate	lamp.heart_rate	Continuous	Yes
Heart Rate Variability	lamp.heartratevariability_sdnn	Continuous	Yes
Respiratory Rate	lamp.respiratory_rate	Continuous	Yes

**Table 2:** Secondary features.

Secondary feature name	References featuring links to documentation	Requires additional parameters for Cortex?
Call Duration	[20]	Yes
Call Number	[21]	Yes
Data Quality	[22]	Yes
Entropy	[23]	No
Cognitive Assessment Results	[24]	Yes
HealthKit Sleep Duration	[25]	Yes
Hometime	[26]	No
Inactive Duration	[27]	No
Nearby Device Count	[28]	No
Screen Duration	[29]	No
Step Count	[30]	Yes
Survey Results	[31]	Yes
Trip Distance	[32]	No
Trip Duration	[33]	No
Call Degree	[34]	No

### Pre–Data Processing Information

#### Overview

The design and function of Cortex are motivated by the volume of data, particularly passive data, that a single user can generate in 2 weeks. Assuming a user captures GPS and accelerometer data from the smartphone, each at a rate of 5 Hz, there will be just >1.2 million data points gathered within 14 days. While lower sampling frequencies are possible, the ability to capture additional sensors and data over months or even years illustrates the size of the data set in question. Cortex is designed to work flexibly with these data. This section reviews its foundations and assumptions to provide key background information to enable easy and effective use of Cortex for broad applications including data processing and data visualization.

#### IDs and Their Hierarchy

Working with the data requires being able to identify users and studies. For every participant that downloads the mindLAMP app, a researcher needs to create a user profile for them in the mindLAMP dashboard. Upon creation of a user profile, mindLAMP will automatically generate a unique ID specific to the patient and the investigator account (researcher ID). The user ID is given in the format of a U followed by 10 digits (eg, U0123456789). These IDs are not to be confused with the researcher ID, which is provided in a 20-character string format of numbers and letters (eg, 123456abcdef78g912hi). Within the investigator mindLAMP account, multiple studies (groups) can also exist. The studies themselves also have a unique ID, following the same format as the researcher ID.

**Figure 2:**
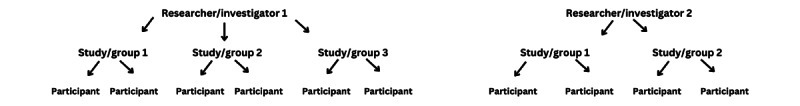
LAMP hierarchy: This figure shows the hierarchy within LAMP. Researchers, also known as investigators, are at the top. Each researcher/investigator can have multiple different studies, and within each of those studies are individual participants.

The utility of having multiple studies under a singular researcher ID is to allow for multiple versions (eg, A/B testing, all sensors active or no sensors active, feedback shared in the app or no feedback shared in the app) of a larger investigation (Figure 2). This can be useful when assessing the impact of features or when seeking to offer participants multiple ways to partake, especially around how much passive data they wish to share. It can also enable different schedules of assessments or app reminders for participants as set by the research team.

#### Epoch Time and Milliseconds

Instead of dates, which can have multiple formats, Cortex uses a modified version of Epoch time. Epoch time is the number of seconds that have passed since January 1, 1970, 12 AM Coordinated Universal Time (UTC) to a specified date and time. The modification Cortex uses is that it uses the number of milliseconds instead of seconds. To provide an example, December 1, 2023, 12 AM is 1,701,450,000,000 in the modified Epoch time. In addition to representing date values in modified Epoch time, all other time-related fields (eg, time duration) will also use this format. For the remainder of the paper, when Epoch time is mentioned, it will be the modified version.

For the sake of convenience to the user, this paper will provide resources to turn dates into Epoch time and helpful variables to change milliseconds to more comprehensive units, such as seconds or days [35]. In addition, many different Epoch time converters on the internet can assist with conversions.

**Figure 3:**
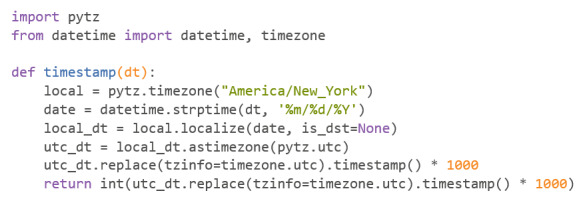
Codebook 1.

Dates can be converted into Epoch time using a Python function combined with various Python packages, such as “pytz” and “datetime” (Figure 3).

**Figure 4:**
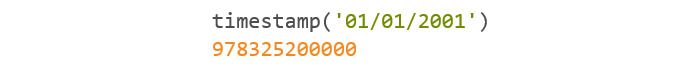
Codebook 2.

This function takes a date in the form of MM/DD/YYYY in a string and converts it into Epoch time. An example of this function in use can be seen in Figure 4.

**Figure 5:**
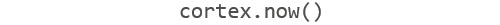
Codebook 3.

Cortex has a useful command that gives the epoch time when the command is run (Figure 5). This command will print the Epoch time if it is not saved into a variable.

**Figure 6:**
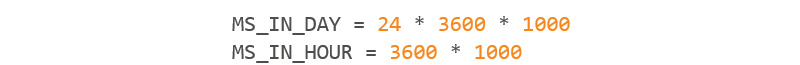
Codebook 4.

Finally, it is good practice to keep variable names that allow for easy conversions of milliseconds such as the variables given in Figure 6.

#### Imports

Cortex is designed to provide users with meaningful and understandable metrics. When combined with various Python packages, it is even more powerful for data processing. Table 3 shows common Python packages that are often useful to run with Cortex. These are the packages that will be used in the rest of the paper and are useful to explore if planning to run Cortex. There are many more Python packages that can be used, and Table 3 should not be viewed as a limiting factor on what can be done with the data processed by Cortex.

**Table 3:** Common Python Packages used to help manage and support data manipulation and analysis.

Package name	Package usability	References featuring the documentation link
Pandas	Allows for data manipulation and simple analysis within DataFrames	[36]
Numpy	Allows for scientific computations in Python	[37]
Datetime	Allows for manipulation of dates and times	[38]
Pytz	Allows cross-platform time zone calculations	[39]
Seaborn	Allows for data visualizations	[40]
Matplotlib	Library for creating visualizations in Python	[41]
Calplot	Used to create heat maps per calendar year by plotting time series data	[42]
Altair	Visualization Library for Python	[43]
Plotly	Python Graphing Library that makes interactive visualizations	[44]

### Basic Use of Cortex

Building on the previous sections, this section explores how to import Cortex, score active data surveys, pull active data, pull passive data, and use several miscellaneous Cortex commands. This section discusses issues related to poor data quality (ie, low engagement from the user and errors in data collection) and how to use these commands to easily assess and troubleshoot both active and passive digital phenotyping data quality issues.

#### Importing Cortex

**Figure 7:**
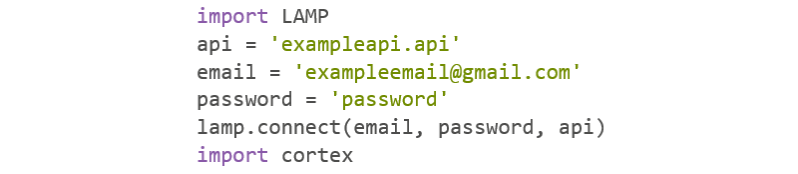
Codebook 5.

Cortex is a Python package that uses the mindLAMP application programming interface (API) to collect data from various users. In order to use its API, the user must have an access key provided by the mindLAMP consortium (Figure 7).

#### Checking Engagement and Data Quality

Participant engagement is critical to any study, especially for digital phenotyping, in which the goal is to collect active and passive data from their smartphones. A lack of active participant engagement limits the amount of data that can be pulled from LAMP, and missing data will reduce the quality of analysis that can be performed [6]. Engagement thus encapsulates (1) a participant’s active participation in taking surveys and cognitive assessments and (2) a participant’s passive data quality, a ratio of actual versus expected smartphone signal (passive) data captured each day. Due to this importance, it is best practice to check engagement and data quality metrics before proceeding with other analyses. Prior research suggests that active engagement is linked to passive data quality; if the app is not actively opened and used for several days, the phone may ignore the app’s request to capture high-frequency passive data [6].

**Figure 8:**
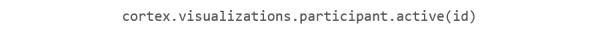
Codebook 6.

To assess participant’s engagement with active data, it is useful to evaluate how many surveys or cognitive assessments have been completed in a certain time frame. Cortex supports such assessments with the command shown in Figure 8.

**Figure 9:**
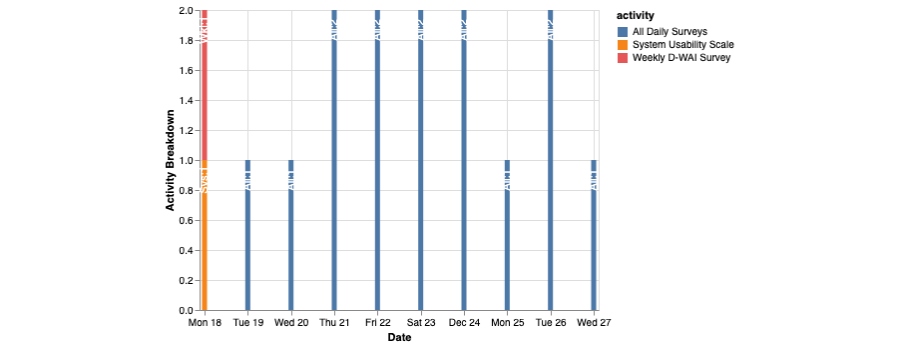
Cortex active data visualization. This figure shows the activities that a participant has completed over time using mindLAMP. The x-axis shows the date of the activities completed, while the y-axis shows how many activities were completed. The color of the bar represents which activity was completed. The blue bars represent a created survey that the participant was supposed to take twice a day. The orange bar represents a survey that the participant completed in regard to the app itself and its ease of use. The red bar is another survey that represents responses to questions on the digital working alliance. D-WAI: Digital Working Alliance Inventory.

This command displays a graph of the activities that the participant completed, with the date of completion as the x-axis (Figure 9). Activities seen can vary from mindfulness exercises to the surveys that the participants complete.

Given that a study can be customized to capture different active and passive data streams at different frequencies, the parameters within the command to check data quality can be changed to suit different purposes. For the scope of the paper, every parameter for every command will not be explained in detail. Instead, the most important parameters will be explained, and a link to the documentation will be provided. The documentation will contain the other parameters not covered in the paper [45]. If a documentation link is not provided, all parameters are covered in detail.

The important parameters of cortex.visualizations. participant.active are explained as follows:

1. id_list: this parameter represents the user for which the researcher is generating the graph. To generate the graph for a single participant, one should make this parameter a Python string with the user ID. This parameter also accepts a list of participant IDs, study IDs, and researcher IDs. If >1 participant ID is provided or represented within a study or researcher ID, multiple graphs will be printed.
2. sample_length: this parameter represents the maximum number of days that can be represented in the generated graph. It is possible to use an arbitrarily large number to pull every single day that a participant has completed a task. The graph will start with the first event completed.
3. attach_graphs: this parameter is a Boolean value (True or False) that, if true, will attach the graphs to the participant’s Portal Tab in the mindLAMP app for which the graph is generated. This enables real-time sharing of results with participants directly via mindLAMP and provides a simple means for return of results.

**Figure 10:**
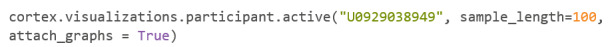
Codebook 7.

An example of this command in use can be seen in Figure 10.

Figure 9 shows the activities of a participant who was asked to complete 2 of the same daily surveys per day. These surveys were named “All Daily Surveys” in mindLAMP and are represented as blue bars as seen in the legend to the right. Looking at the graph, the participant has been active in terms of their engagement with surveys and has completed at least 1 survey between December 19 and December 27. The participant completed the task of taking the survey twice on December 21, 22, 23, 24, and 26.

**Figure 11:**
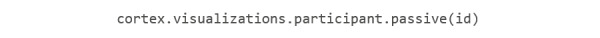
Codebook 8.

The command used to assess passive data quality is similar to that used to assess active data as shown in Figure 11.

This command generates 4 graphs per participant. Two of the graphs represent the amount of data coming from the GPS sensor. The other 2 graphs will represent the amount of data coming from the accelerometer sensor. The graphs generated will be either a scatterplot or a heat map. Each of these 2 graphs will have a representation of the “ideal” amount of data being received. For the scatterplot, a horizontal red line is placed. For the ideal amount of data collected, the scatter plot should be above the line. In terms of the heat map, a blue star will represent the ideal amount of data collected. These “ideal” amounts should only be used as a reference point. The true ideal amount of data will always be up to the researchers and set by the study.

The important parameters of cortex.visualizations. participant.passive are explained as follows [45]:

1. id_list: this parameter represents the user for which the researcher is generating the graph. To generate the graph for a single participant, make this parameter a Python string with the user ID. This parameter also accepts a list of participant IDs, study IDs, and researcher IDs. If >1 participant ID is provided or represented within a study or researcher ID, multiple graphs will be printed.
2. sample_length: this parameter represents how many days the user wants to query for. Unlike the sample_length parameter in cortex.visualizations.participant.active, the user cannot use an arbitrarily large number to query for all the data from a participant. If a user does take this approach, they will end up querying for days that a participant was not collecting data, causing the scale of the axes to be off and creating unnecessary blank space on the heat map.
3. days_ago: this parameter represents where the user wants the end of the x-axis to be.
4. reset: this parameter is a Boolean value (True or False) that will reset any previous graphs generated for the participant. If a graph has already been generated, any new information being queried for will be added to the existing graph. If the user does not want this, use reset=True.

**Figure 12:**
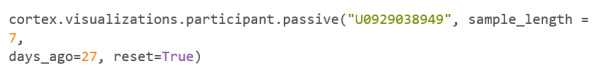
Codebook 9.

An example of this command in use can be found in Figure 12. In this example, the researcher wanted to query for the first week of the participant’s time during a study. The researcher knows that the participant consented to data collection 1 month ago (31 days). The user also had generated a previous graph and wanted to reset the existing graph.

**Figure 13:**
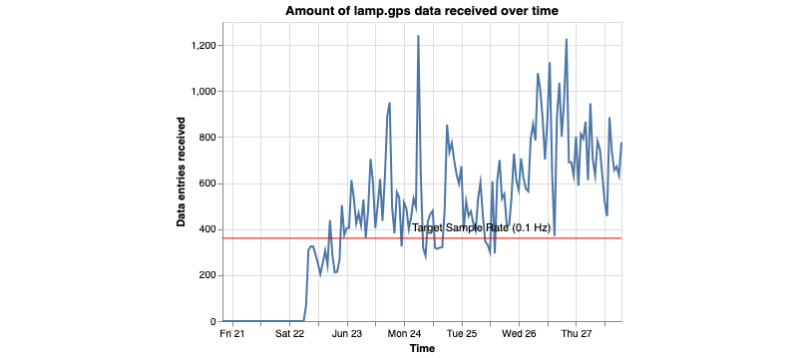
This figure shows how many GPS data points are being collected over time. The x-axis represents the time of data point collection, and the y-axis represents the amount. Ideally, the user would want to see the blue line never drop below the red.

**Figure 14:**
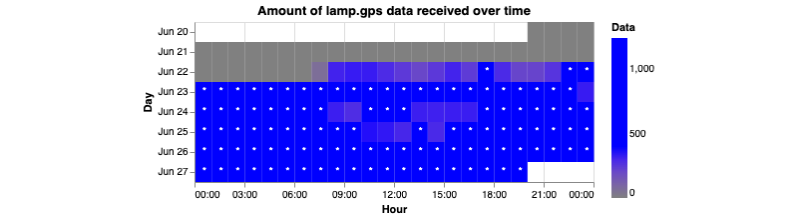
This figure shows how many GPS data points are being collected over time. The beginning of the x-axis represents the very start of the day, 12 AM or 00:00 in 24-hour time. Each hour is represented as a square and given a gradient of blue, with the deeper blues representing more data points collected. Each row represents 1 day and starts at the top. Ideally, the user would want each square to be blue with a little star within.

**Figure 15:**
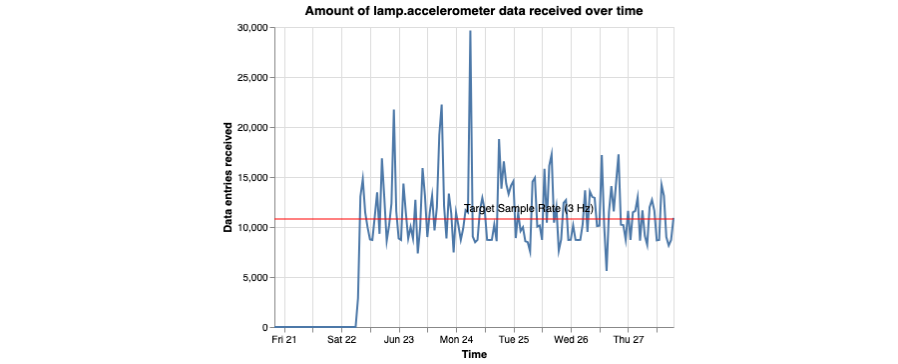
This figure shows how many accelerometer data points are being collected over time. The x-axis represents the time of data point collection, and the y-axis represents the amount. Ideally, the user would want to see the blue line never drop below the red.

**Figure 16:**
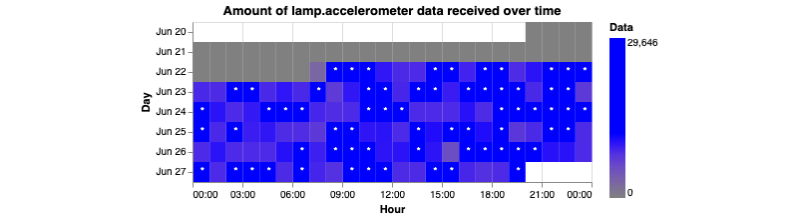
This figure shows how many accelerometer data points are being collected over time. The beginning of the x-axis represents the very start of the day, 12 AM or 00:00 in 24-hour time. Each hour is represented as a square and given a gradient of blue, with the deeper blues representing more data points collected. Each row represents 1 day and starts at the top. Ideally, the user would want each square to be blue with a little star within.

The examples shown in Figures 13-16 highlight that LAMP is collecting passive data of accelerometer and GPS for this single user, although on some days, the data quality captured is below the target. While beyond the immediate scope of this paper, possible reasons include the phone being in low-power or airplane mode, lack of active engagement (which was assessed at the beginning of this section), or technical errors (one common error being the participant’s device being on low-power mode). Monitoring passive data quality is easy and can be done on a weekly or even daily basis to rapidly spot issues and help a study or patient troubleshoot.

#### Activity Events

Surveys and sensors are not the only types of data that can be captured. mindLAMP also offers functionality beyond active and passive data collection to enable users to engage with psychoeducation (the L in LAMP) and complete therapeutic exercises (the M in LAMP). For some studies, this can be important or even the focus of the research question. For example, “Does the efficacy of completing mindfulness on mindLAMP vary by location or does cognitive therapy work better on nights with more sleep duration?” To accomplish this from a data perspective, the user can access information regarding everything a participant engages in within mindLAMP. Every time a participant completes an activity within mindLAMP, details about the event are recorded. These details include when the activity was started, how long it took, and specific details relating to the activity. The specific details can include the answer to a question within a survey, the results of a cognitive assessment, or the time stamp when a mindfulness exercise was completed.

**Figure 17:**
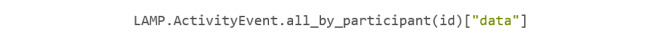
Codebook 10.

A researcher or clinician can create their own activities, including custom surveys, custom psychoeducation, and custom therapeutic activities. To ensure that these custom activities can be easily scored and work well with Cortex, there is a standard data format that is important to understand when seeking data from any activity event. In order to view these activity events for any study, this Cortex command, which can be seen in Figure 17, can be used.

The command will print a Python dictionary that contains every activity a participant has completed. The dictionary will also contain information related to the activity being completed, which will be contained within “temporal_slices,” a key within the dictionary (or column within a DataFrame). For example, if a participant took a survey, every question within the survey would be contained within “temporal_slices.’’

**Figure 18:**
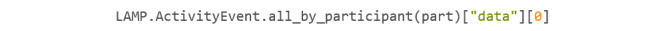
Codebook 11.

An example of the command in use can be found in Figure 18.

**Figure 19:**
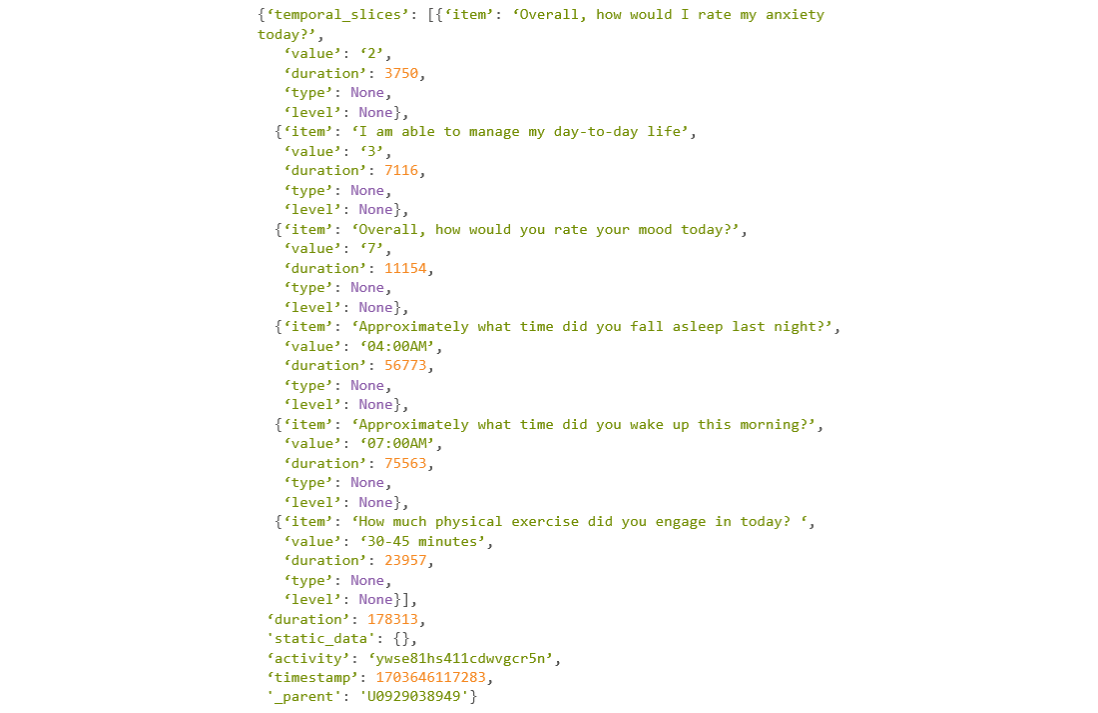
Codebook 12.

Since this command will print every activity completed, it can have a very long output. In the example (Figure 19), only the most recent activity completed will be printed.

Going through this output, it can be seen that there are 6 keys within the Python dictionary, each describing something specific about the activity that was completed. Starting at the top in the “temporal_slices” key, the information contained within is specific details about the activity event and actions within. In this example (Figure 19), since the activity event was a survey, each answer to the questions is recorded, along with how long it took to answer the question. The next key in the dictionary is “duration.” This is simply how long the entire activity event took. The “static_data” key is empty in this activity. Static data refer to the details about certain activity events, specifically the games offered within mindLAMP. These details include points scored, correct answers, attempts, and so on. Moving to the next key, “activity” represents the activity event that was completed. Each activity event is given a string for identification regardless of activity type (eg, 1 survey will have a different identification string from another). Within this example, the activity completed was “All Daily Surveys.” The final 2 keys are “timestamp” and “_parent,” which are the time the activity was completed and who completed the activity, respectively.

#### Survey Grading

Surveys, which are a common form of active data collection, can be completed on the LAMP platform and scored with Cortex. Scoring with Cortex is an efficient way of analyzing the results of the surveys and prevents mistakes caused by calculating scores by hand.

To score a survey using Cortex, a Python dictionary needs to be created. These dictionaries provide Cortex with instructions on how the user wants the survey to be scored. For example, a participant may have completed a daily survey that asked them 3 questions. The 3 questions being “Overall, how would you rate your mood today?” “Overall, how would I rate my anxiety today?” and “How much physical exercise did you engage in today?” relating to the participant’s mood, anxiety, and exercise level, respectively.

**Figure 20:**
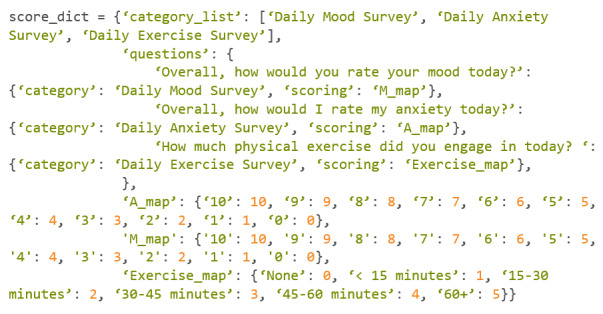
Codebook 13.

This scoring dictionary can be seen in Figure 20, with scoring parameters easily adjusted by the researcher.

This dictionary can be updated with any survey that has been taken, but the dictionary can only take numeric outputs, whether that be a Boolean (True or False) or an integer.

With a start time, end time, and scoring dictionary, the user can query for scores. The scores will be labeled to match how categories are named, in this case they will be labeled “Daily Mood Survey,” “Daily Anxiety Survey,” and “Daily Exercise Survey.”

**Figure 21:**
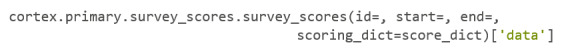
Codebook 14.

The command to grade the survey can be seen in Figure 21.

The important parameters of cortex.primary.survey_ scores.survey_scores are explained as follows:

1. id: this parameter represents the participant’s survey that is being graded. Unlike the Cortex visualizations, ID is limited to 1 participant at a time. If the user wants to grade multiple participants at once, consider a loop. In addition, only putting the user ID without the parameter title (id=) will cause an error.
2. start: this parameter represents the start of the time period the user wants to query for (epoch time).
3. end: this parameter represents the end of the time period the user wants to query for (epoch time).
4. scoring_dict: this parameter represents the scoring dictionary that was created in Figure 20.
5. return_ind_ques (optional): this parameter is a Boolean value (True or False) that, if True, will return the individual question along with the results of the survey. Since this parameter is optional, if it is not included within the command, Cortex assumes it to be false.

**Figure 22:**
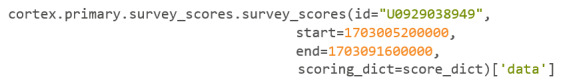
Codebook 15.

An example of this command can be found in Figure 22.

**Figure 23:**
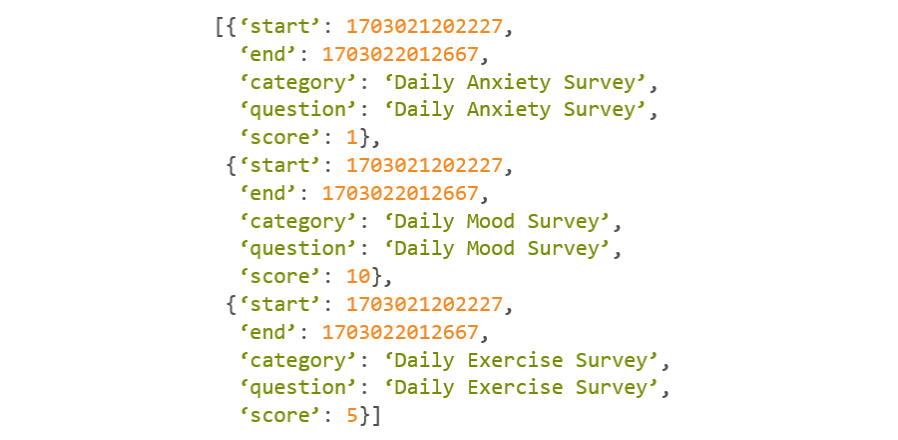
Codebook 16.

In this example, the researcher wanted to query every survey taken from December 19, 2023, to December 20, 2023. The researcher also did not want Cortex to return the individual questions within the surveys (Figure 23).

#### Passive Data Processing

**Figure 24:**
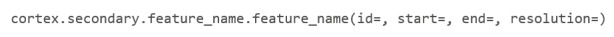
Codebook 17.

Passive data streams (eg, accelerometers) are often the most useful when that data can be transformed into behavioral features. Cortex has 2 ways of processing the raw passive data collected from the LAMP platform. The first way a user can process passive data is individually, through the format of the command shown in Figure 24.

The important parameters of cortex.secondary.feature_ name.feature_name are explained as follows:

1. feature_name: this parameter is in the name of the command. It represents what passive data feature the user wants to query for (refer to Table 2 for the list of feature_names that are currently available).
2. id: this parameter represents the participant that the user wants to query for.
3. start: this parameter represents the start of the time period the user wants to query for (epoch time).
4. end: this parameter represents the end of the time period the user wants to query for (epoch time).
5. resolution: this parameter represents the time frame of which the passive data feature is calculated in milliseconds. For example, if the user wanted to calculate hometime in A DAY, the user would have a resolution equal to the number of milliseconds in a day.
6. The parameters above are required for ALL features, but some features require more parameters. Cortex will produce an error if the required parameters are not included while executing the command. The features that require more than the parameters above can be seen in Table 2.

**Figure 25:**
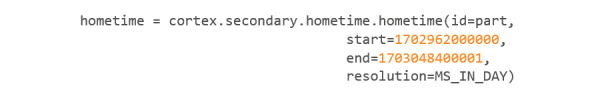
Codebook 18.

An example of this command can be found in Figure 25.

**Figure 26:**
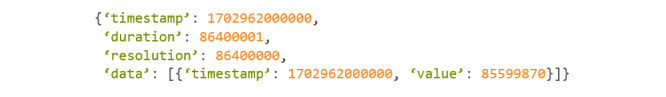
Codebook 19.

With this command, the user is looking for how much time the participant spent at home from December 19, 2023, to December 20, 2023, specifically 12 AM, for both dates. Since the resolution is binned by the number of milliseconds in a day, Cortex will return the amount of time at home per day. If the user changed the resolution to the number of milliseconds in a week, Cortex would return the amount of time at home that week. It is important to note that the difference between the start and end time needs to be greater than the resolution by at least 1 millisecond. This is why, in the example, the end time ends with a 1 (Figure 26).

**Figure 27:**
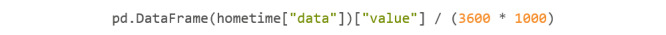
Codebook 20.

Looking at the results, it can be seen that this participant spent 85,599,870 milliseconds at home, but for the sake of understanding, this value should be changed into something more digestible. This can be seen in the example continuation in Figure 27.

**Figure 28:**
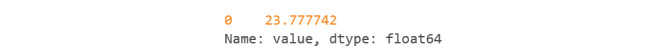
Codebook 21.

This command uses the pandas package to take the “value,” which is the amount of home time in milliseconds and converts it into hours (Figure 28).

Within this example, it can be seen that this participant has spent nearly the entire day at home.

**Figure 29:**
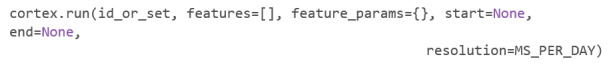
Codebook 22.

The second way that Cortex can process sensor data is with the cortex.run() command. The functionality of the parameters is largely the same as that of the cortex.secondary (Figure 29).

Cortex.run is meant to be customizable. Every parameter, from ID to the start and end times, is meant to give all the information one needs in one go. Going through the parameters, examples of what can be customized are given as follows (rest of the parameters are given elsewhere) [46]:

1. id_or_set: this parameter is who the user is querying for. The user can run this command with 1 participant, many participants in a contained list, or a study or researcher ID.
2. features: this parameter describes what to query for (refer to Table 2 for the features that are available to be queried).
3. start: this parameter represents the start time in epoch time.
4. end: this parameter represents the end time in epoch time.
5. resolution: this parameter represents the time frame of which the passive data feature is calculated in milliseconds. For example, if the user wanted to calculate hometime in A DAY, the user would have a resolution equal to the number of milliseconds in a day.

**Figure 30:**
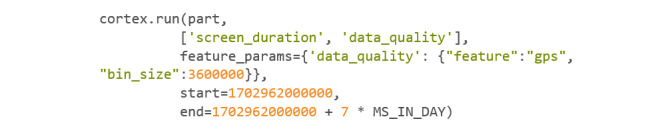
Codebook 23.

An example of this command in use can be found in Figure 30.

**Figure 31:**
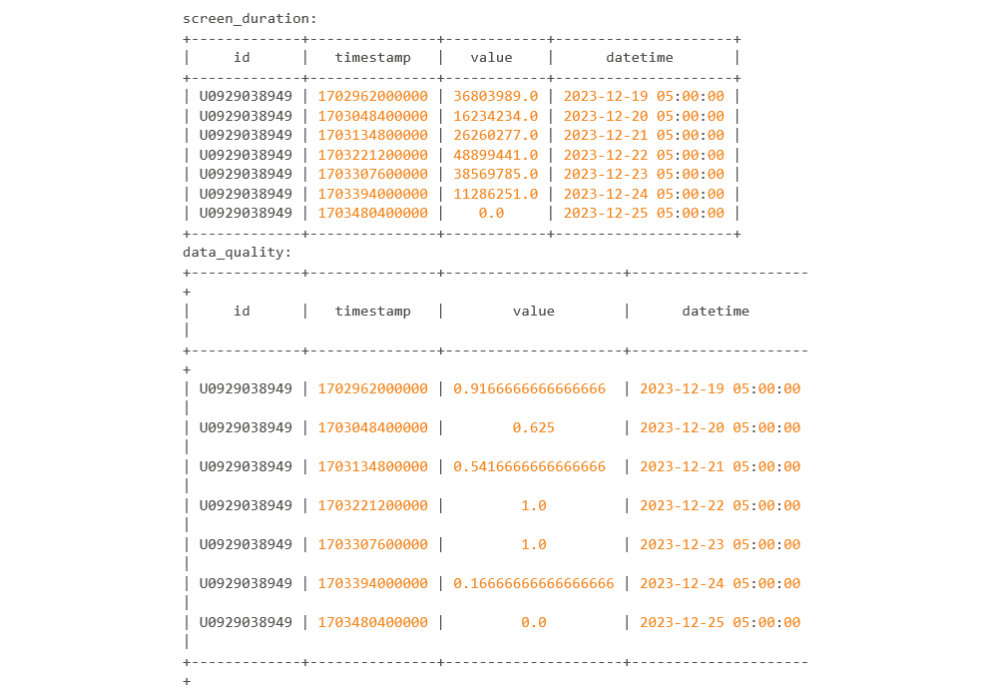
Codebook 24.

In this example, the researcher wanted to query for both screen_duration and data_quality for 1 week. Within the feature_params parameter, it is specified that data quality will be calculated using the GPS sensor and not the accelerometer. The bin size is set at 3,600,000, which means that Cortex will split the time between the start and end time into “chunks” of 3,600,000 milliseconds, which is the equivalent to 1 hour. The value calculated under data_quality is the percentage of bins or “chunks” that have a point of either GPS or accelerometer data. In this case it is GPS data. The results below are cleaned up from the normal result using the “tabulate” Python package and provide a visually appealing table (Figure 31).

**Figure 32:**
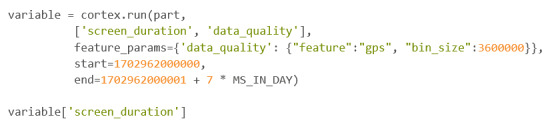
Codebook 25.

The actual code chunk will print a data dictionary, which can be confusing to read; thus, it is recommended that the user save it as a variable when using cortex.run. Once it is saved as a variable, the user can call individual keys from the dictionary. An example of this can be seen in Figure 32.

**Figure 33:**
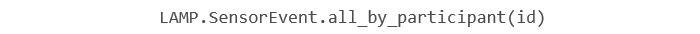
Codebook 26.

Researchers also can pull raw sensor data. The functionality of this command is much like pulling activity events, which can be referred to in the *Activity Events* section. The command can be run without parameters but will return an incomprehensible amount of data (Figure 33).

Parameters can be used within this command to narrow how much data are received and what sensor spec to return:

1. id: this parameter represents the participant that the user wants to query for.
2. origin: this parameter represents what sensor spec the user wants to query for (refer
  Table 1
  for the features that are available to be queried).
3. _from: this parameter represents the start time in epoch time.
4. to: this parameter represents the end time in epoch time.
5. _limit: this parameter represents the maximum amount of data points to pull.

**Figure 34:**
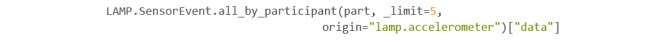
Codebook 27.

An example of this command in use can be seen in Figure 34.

In this example, the researcher wanted to query the first 5 accelerometer data points for the participant.

**Figure 35:**
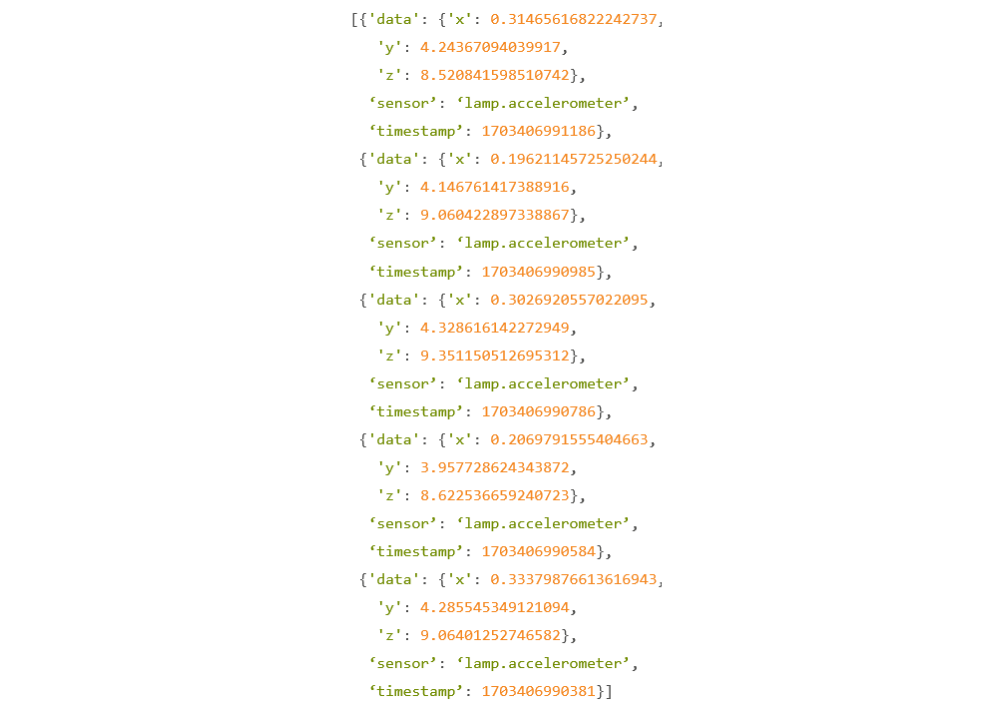
Codebook 28.

Looking at the example (Figure 35), it can be seen what raw data looks like, and it highlights the necessity for data pipelines such as Cortex.

With the information present thus far, the reader is able to pull both active and passive data. With this background, the paper will now shift into data manipulation and how to create data visualizations that can give the user insight into participant’s behavior in relation to data gathered.

### Advanced Participant Level Example and Data Manipulation

#### Overview

Cortex makes pulling data simple, but most of the time, the data format given is not compatible with various Python packages for creating charts, tables, and different machine learning techniques. Due to this difficulty, it is worthwhile to consider an example where various levels of data manipulation are used to create a pandas DataFrame that will allow for Python packages to be used. The subsequent subheadings (or sections) will elaborate (1) grading surveys and pulling passive data for a participant, (2) how to convert time stamps in epoch time into a date format, (3) splitting DataFrames, (4) cleaning DataFrames, (5) merging of DataFrames and final cleanup using pandas, (6) creating the correlation matrix and making it visually appealing, and (7) verifying and gathering more information.

While many types of data visualization are possible, 1 compelling data visualization that is often used is a correlation matrix. These matrices can point the user in the right direction in terms of understanding the relationships between active and passive data. However, it is important to understand that although variables may correlate with each other, there may be confounding variables that are not represented. To combat this, extra examples will be given to implement scientific processes and give weight to what the data are telling the user.

This example will go through each step to create the correlation matrix. These steps will also be labeled in reference to the enumerated list above for easy access.

Within the correlation matrix, the variables that will be included are (1) anxiety (measured daily through surveys on a scale of 1-10), (2) mood (measured daily through surveys on a scale of 1-10), (3) exercise length (measured daily through surveys; scale is a range of durations up to 60 min), (4) screen duration (collected from passive data sensors), (5) step count (collected from passive data sensors), and (6) data quality (calculated using passive data sensors).

#### Grading Surveys and Pulling Passive Data for a Participant

**Figure 36:**
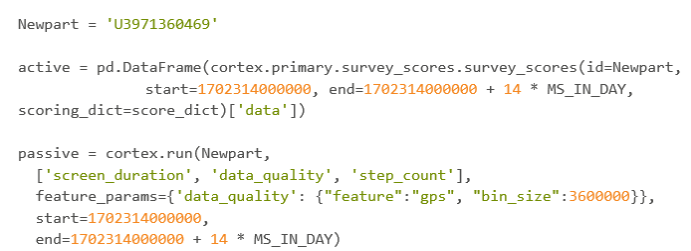
Codebook 29.

After importing packages and connecting them to the API, the user should specify the user ID and save it within a variable. in this example, “Newpart.” The user should then pull the active and passive data using the respective Cortex commands and save the results within properly named variables (Figure 36).

The dictionary used to grade the surveys is the same dictionary used within the *Survey Grading* subsection.

#### Converting Time Stamps

**Figure 37:**
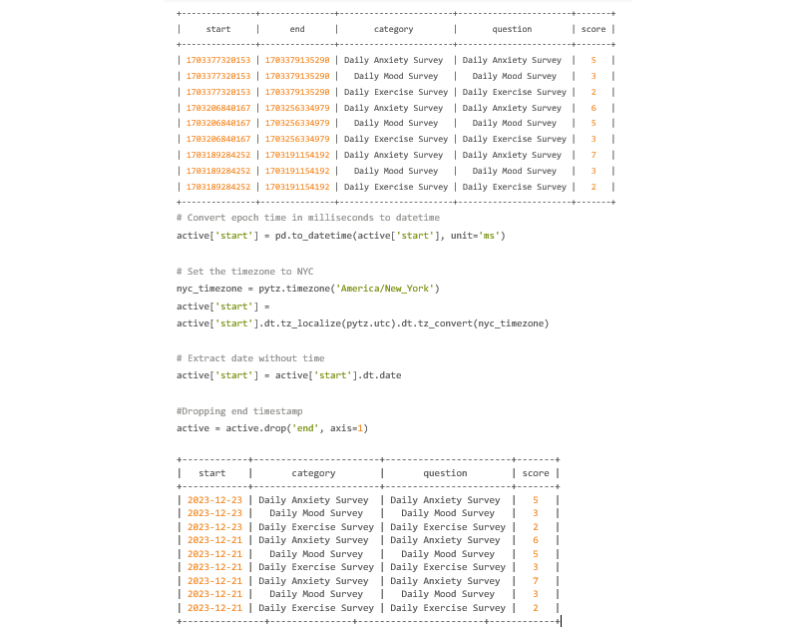
Codebook 30.

Taking a look at the “active” variable, the start time stamp needs to be converted from epoch time, which is in UTC, to a date in EST. Doing this will allow the user to merge the passive and active DataFrames on the formatted date. Knowing that the surveys started and ended on the same date, only the start time stamp needs to be converted. The end time stamp will be deleted. Figure 37 shows the current state of the active variable before time stamp conversion.

**Figure 38:**
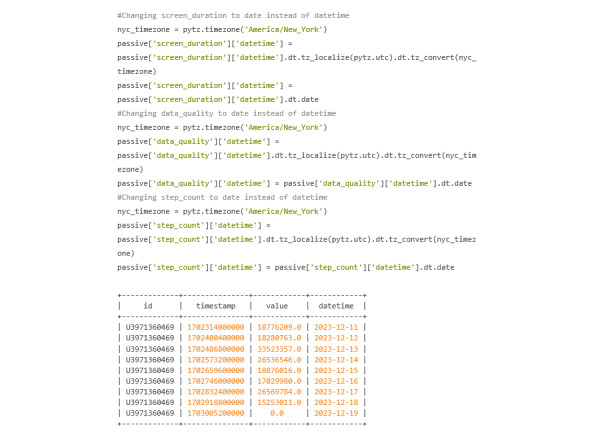
Codebook 31.

The passive data do not need as many steps because the cortex.run produces a datetime value. The user does need to be wary that this datetime value is in the UTC time zone and needs to be converted (Figure 38).

Screen_duration is currently contained within the “passive” dictionary. Though only screen_duration is shown, both data_quality and step_count are also contained within “passive” and look identical to screen_duration in terms of format. Figure 38 shows the first 9 rows of screen_duration within the passive dictionary.

#### Splitting DataFrames

**Figure 39:**
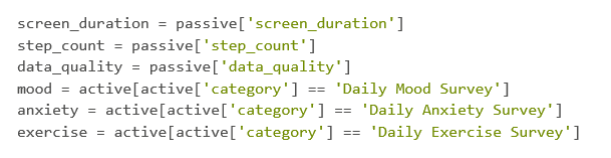
Codebook 32.

For the sake of simplicity in merging and reducing redundancy, the separation of each variable allows for precise changes to be made (Figure 39).

#### Cleaning DataFrames Using Pandas

**Figure 40:**
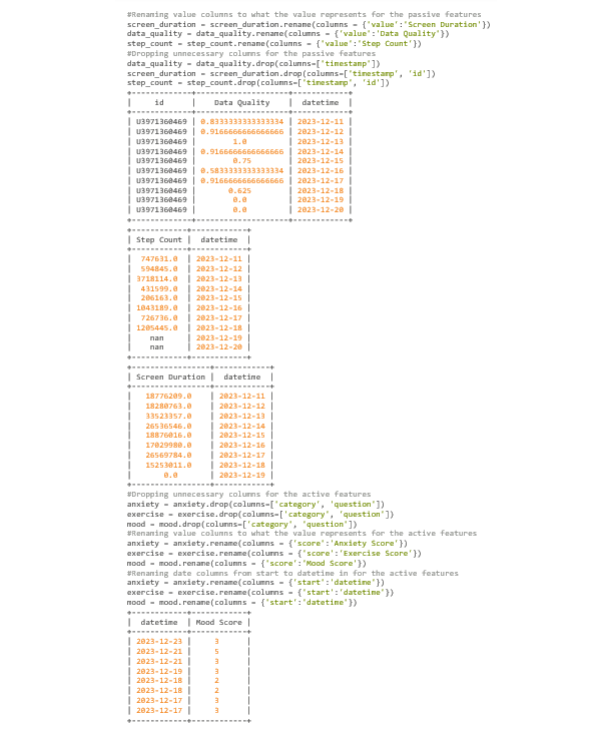
Codebook 33.

When cleaning data and preparing it for merging, it makes sense to have a plan regarding what the user wants the DataFrame to look like. This can help to prevent unnecessary commands. In this example, the final DataFrame will have a column for the UserID, a date that will be merged, and 1 column per data feature. Currently, the passive DataFrames have the most information in regard to the columns, but only 1 of the 3 needs more than just the date and its respective data. Knowing this, the data_quality DataFrame will be established as the base and will be the only DataFrame that keeps its UserID column. It is also worth noting that each of the “value” columns, which are within each of the DataFrames, will need to be renamed into what the value represents. For instance, for the step_count DataFrame, the “value” column needs to be renamed into “stepcount” or some variation of that (Figure 40).

**Figure 41:**
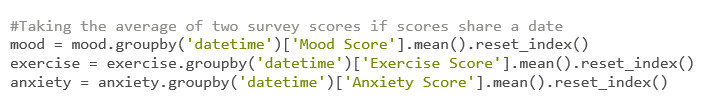
Codebook 34.

Notice how in the DataFrames shown in Figure 40, there are multiple instances of the same date that have a score. This is because this participant took >1 survey in a day. There are many different solutions to remedy this, but for this example, the average between the scores will be taken (Figure 41).

#### Merging of DataFrames and Final Cleanup

**Figure 42:**
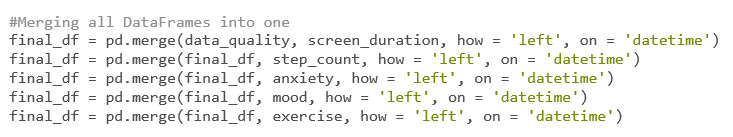
Codebook 35.

Now that every data point is attached to its formatted date (Figure 42), each of the DataFrames is ready to be merged.

Now that the DataFrames are merged (Figure 42), the user can look for discrepancies. Two glaring edits that need to be implemented into this DataFrame are switching the locations of “Data Quality” and “datetime” and changing the units of “Screen Duration” from milliseconds to hours.

A keen eye will also notice strange results within the “Step Count” column. It is known that the values within “Step Count” are supposed to represent how many steps a participant has taken within a day, but the values obtained are way too high to be accurate. It is unknown what went wrong while collecting this participant’s data, but it goes to show that researchers should keep a sharp eye out for discrepancies. For this example, the step count correlations will be taken with a grain of salt.

**Figure 43:**
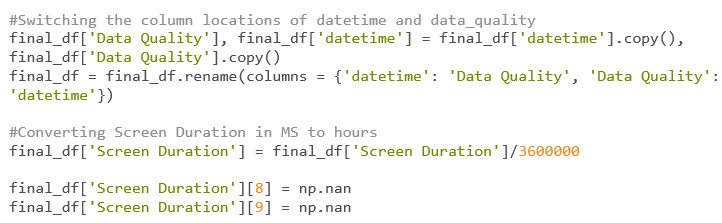
Codebook 36.

Finally, on December 19 and 20, the screen duration is 0 for both. Since 0 can and will be used within correlations, the result can be skewed by outliers. Due to this fact, it is reasonable to turn these *0’*s into NaN values using the Numpy Python package (Figure 43).

#### Creating the Correlation Matrix and Making It Visually Appealing

**Figure 44:**
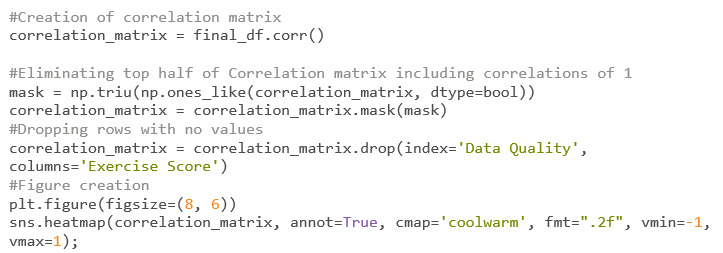
Codebook 37.

**Figure 45:**
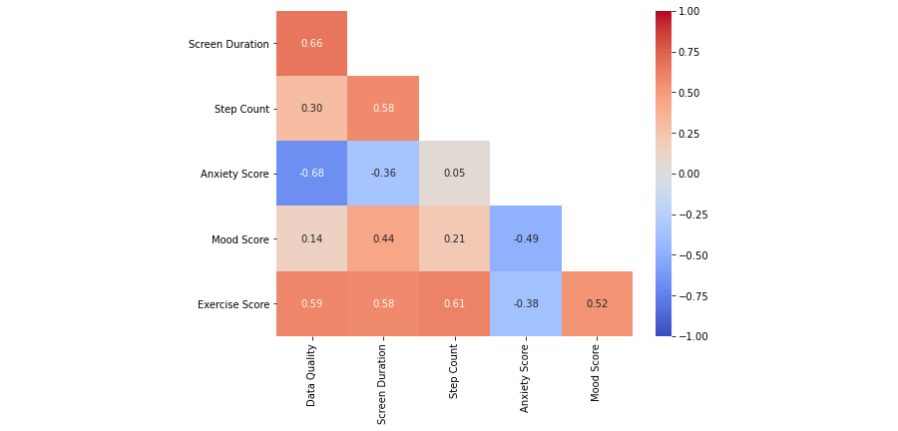
Correlation matrix. This graph shows each variable and its relationship to the other variables within the final DataFrame. Correlations closer to 1 represent a positive linear correlation, whereas correlations close to negative 1 represent a negative linear correlation. Positive in this context means that both variables are going in the same direction. Negative means that the variables are heading in opposite directions.

The correlation matrix (Figure 44) is created with a simple command using the “pandas” Python package. To make it visually appealing, the “seaborn” package will be used. The result is shown in Figure 45.

Looking at the graph, it is possible to see an overview of how the variables may correlate with each other. It is important to note not only that correlation does not equal causation but also how this graph can help share back both active and passive data with participants. Scatterplots are also helpful in obtaining an alternative image of what these data may represent.

**Figure 46:**
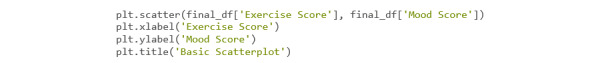
Codebook 38.

#### Verifying and Gathering More Information

**Figure 47:**
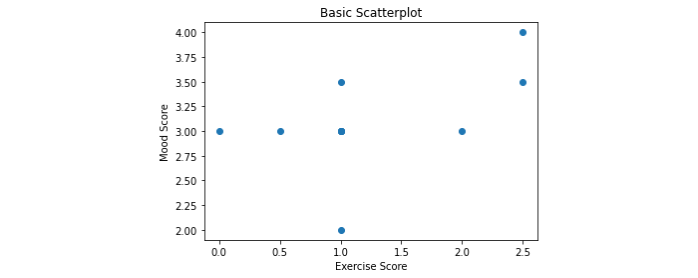
Scatterplot of exercise score versus mood score. This graph shows the mood score with respect to the exercise score. The x-axis represents the exercise score, with higher scores meaning the participant completed more exercise that day. The y-axis represents the mood score, with higher scores representing a more positive mood. Each dot represents 1 day.

In the correlation matrix shown in Figure 45, there are some interesting correlations to look at. Perhaps the most interesting is the 0.52 correlation between exercise score and mood. The assumption that most people would make is that if the exercise score is higher, the participant’s mood would also be higher, but to ensure this is the case, a scatter plot should be made (Figure 46). The scatter plot results are shown in Figure 47.

Looking at the graph, it can be seen that the initial assumption that mood improves as the exercise score increases looks to be correct. For the truly diligent user, more information can be gathered by running a simple linear regression, especially due to the linear look of the data points. To do this, a Python package named “statsmodels.api” will be used.

**Figure 48:**
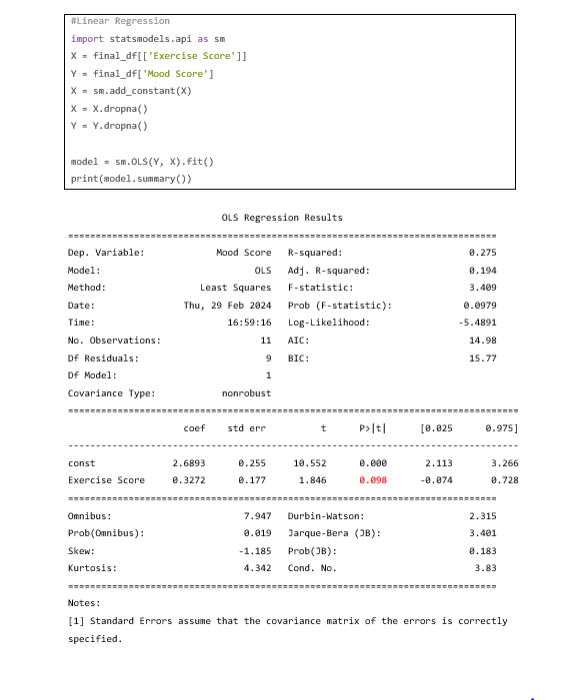
Codebook 39.

Looking at the *P* value in red in Figure 48, it can be seen that this relationship is approaching the point where it would be considered significant (*P*<.05) This is not to say that this is not a valuable result. There absolutely is a case to be made that this participant’s mood can benefit from increased exercise.

## Discussion

### Overview

**Figure 49:**
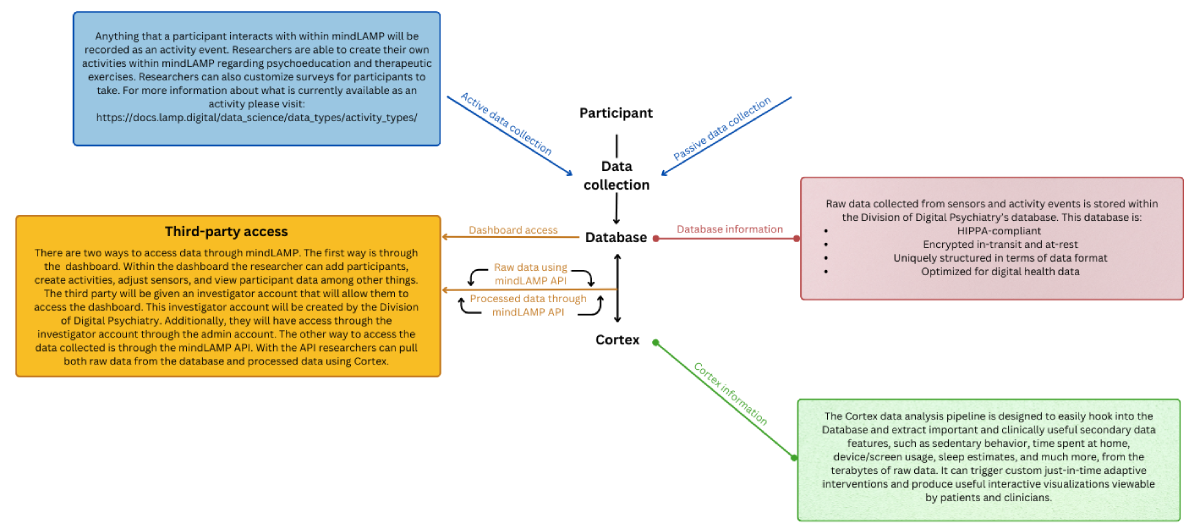
A schematic of a common deployment of mindLAMP, hosted by the Beth Israel Deaconess Medical Center team and Cortex. API: application programming interface. HIPAA: Health Insurance Portability and Accountability Act.

Thus far, this paper has illustrated the technical process of using Cortex to process, manipulate, visualize, and analyze digital phenotyping data. To more clearly illustrate the utility of these functionalities, we will discuss below the significance of the steps outlined in this paper, as well as use cases. Figure 49 highlights a common workflow with Cortex that is applicable to many types of studies and use cases.

The basic features of Cortex can be immediately translated into analysis of digital phenotyping data. For example, Cortex collects time stamps not only of the start and end time of active data activities (eg, a survey) but also of the time of specific item responses for a given survey and of any discrete passive data points. Prior research has used analytical methods such as this to predict self-reported thoughts of harm based on latency [47] in responding to items assessing such thoughts. Furthermore, epoch time provides foundational support for pivotal features that can be derived from Cortex, such as sleep duration and screen use data, which allow the transformation of passive data into clinically relevant variables. While discrete data points can be informative, even without incorporating time (eg, an individual’s home may be approximated for most by the GPS point most frequently visited), the formatting of data points with time stamps allows for more nuanced analysis and deeper insight. By combining basic Cortex features, it is also possible to derive novel digital biomarkers as well as replicate those created by other teams.

The nature of digital phenotyping data, when combined with Cortex, is the flexibility in using derived features or even creating novel digital biomarkers. For example, Cortex-derived features, such as sleep duration, time spent at home, screen time, and data quality, have been demonstrated when used in research applying anomaly detection. Specifically, anomalies within personal baselines of active data and Cortex-derived features of data for individuals with schizophrenia have been used to predict clinical relapse, an example of advanced data processing [48]. Using the methods outlined above in this paper, any digital phenotyping data can be processed in features and DataFrames that will enable easy use of the code [49] for anomaly detection. Related applications may include using the digital phenotyping data features and anomaly detection to detect early warning of risk of self-harm, relapse in bipolar disorder, or medication adherence as 3 broad examples.

The nature of digital phenotyping is also well suited when combined with Cortex for machine learning research. Linear regression models are a common tool for analyzing digital phenotyping data, and the readily accessible Cortex features, particularly those derived from passive data, make Cortex a productive API for analyzing the relationships between digital phenotyping–derived features. In addition, the abundance of time and duration columns in the data set makes Cortex a useful tool to prepare data for time series analysis. Recent research has leveraged Cortex to pull data, split it using a windowing approach, and train a variety of Recurrent Neural Network models using a diverse architecture including Long Short-Term Memory. Cortex’s unique strengths in overtime data allow researchers to use the library for a variety of predictive tasks ranging from prognosis prediction to quantitative score prediction.

**Figure 50:**
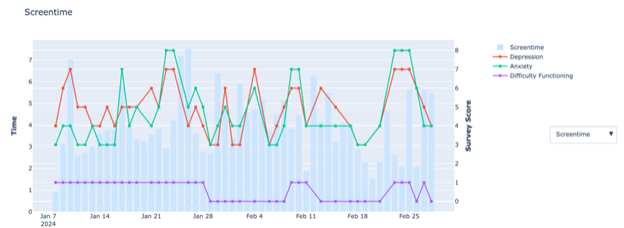
An example of test account data used to illustrate how 1 passive data feature screen time can be visualized in light of different symptoms and functioning metrics.

The utility features such as visualizations for monitoring data quality and providing data back to users have been illustrated in our Beth Israel Deaconess Medical Center–based clinic using mindLAMP [50]. As illustrated above, patients can be offered heat maps of passive data volume generated daily (see the *Checking Engagement and Data Quality* section) in addition to visualizations of active data. With staff allocated to assess patient data quality multiple times per week, they can reach out to patients about their data quality, with specific strategies for ameliorating poor data quality [6]. For example, low passive data quality across sensors may be a result of low weekly app use, as many devices limit app data collection based on use. However, poor GPS quality with high accelerometer quality may simply indicate a misconfiguration of phone settings. The root cause is easy to detect with data visualization. Similarly, patients provided with visualizations of their own active data overlaid with passive data streams can gain insight by observing and even interacting with their own data, as shown in Figure 50.

### Conclusions

Digital phenotyping presents researchers, clinicians, and patients with a wealth of new data. Packages such as Cortex ensure that these data and resulting insights are accessible to all. By reviewing how to pull active and passive data, check the participants’ data quality, manipulate DataFrames, and create basic data visualizations, this paper serves as an interactive guide for advancing reproducible and replicable digital phenotyping research.

Similar to many other tools, Cortex has its limits. While Cortex has been optimized for mindLAMP, it will still work on any data set that meets the formatting requirements outlined in the *Review of Digital Phenotyping Data Types* section. Cortex will attempt to provide meaningful insights and features derived from low data quality, but the quality of such features is questionable. Fortunately, low data quality can be prevented with regular checkups with participants using Cortex for quality checks, as discussed in the *Checking Engagement and Data Quality* section. Clinical judgment is always necessary when interpreting any output, and at the time of this writing, for psychiatry, there are no digital biomarkers recognized by the Food and Drug Administration or other regulatory bodies.

Cortex is an open-source package. As such, it is always evolving, both internally within the team and externally from users worldwide. Due to this evolving nature, this paper is not meant to be rigid but more of a living document to be updated periodically. Updates are posted on the web [51].

## Abbreviations

API: application programming interface
UTC: Coordinated Universal Time

## Data Availability

The data sets generated during and analyzed during this study are available from the corresponding author on reasonable request.
